# Dendritic Cells, Viruses, and the Development of Atopic Disease

**DOI:** 10.1155/2012/936870

**Published:** 2012-10-15

**Authors:** Jonathan S. Tam, Mitchell H. Grayson

**Affiliations:** Section of Allergy and Immunology, Department of Pediatrics, MACC Fund Research Center, Medical College of Wisconsin, Room 5064, 8701 Watertown Plank Road, Milwaukee, WI 53226, USA

## Abstract

Dendritic cells are important residents of the lung environment. They have been associated with asthma and other inflammatory diseases of the airways. In addition to their antigen-presenting functions, dendritic cells have the ability to modulate the lung environment to promote atopic disease. While it has long been known that respiratory viral infections associate with the development and exacerbation of atopic diseases, the exact mechanisms have been unclear. Recent studies have begun to show the critical importance of the dendritic cell in this process. This paper focuses on these data demonstrating how different populations of dendritic cells are capable of bridging the adaptive and innate immune systems, ultimately leading to the translation of viral illness into atopic disease.

## 1. Introduction

Asthma and atopic diseases continue to increase in prevalence in westernized countries [1]; however, the cause for this rise remains unknown. While many hypotheses have been proposed, research continues into the mechanism(s) that drive the development of atopic disease. One potential hypothesis that has been proposed is that viral infections drive the development of atopy and atopic disease [2]. This may be somewhat counterintuitive, as antiviral immune responses are primarily interferon mediated and viewed as a prototypical T_H_1 immune response. Nonetheless, it is well documented that viral infections can aggravate asthma and induce development of asthma and atopic disease, that is, classic T_H_2 diseases. Dendritic cells (DCs), with their unique position bridging the adaptive and innate immune systems, are key resident immune cells in the interplay between viral infections and atopic disease. DCs are crucial to the initiation of the adaptive immune response and are uniquely able to condition Th differentiation. This paper focuses on data supporting a connection between viruses, IgE, and atopy and the critical importance of resident lung dendritic cells in translating a viral illness into atopic disease. 

## 2. Viruses, IgE, and Atopy

Shortly after the discovery of IgE, it was noted that acute viral infections could drive IgE production—both virus specific and nonspecific [3–6]. Although a correlation was noted, the mechanistic reason for this increase (or for the role of IgE in viral illnesses, for that matter) remained unknown. Nonetheless, the association of IgE elevation and the similarity of symptoms between a viral upper respiratory tract infection and allergic rhinitis led some investigators to suggest a possible causative link between viral infections and atopic disease. The first study to hint at this was published in 1979, when Frick et al. [7] studied 13 children from atopic families to see if and when they developed atopic disease. In this small study, 11 of the children were noted to have upper respiratory tract infections in the 1 to 2 months prior to initial development of allergic sensitization. Because of this association, the authors hypothesized that viral infections might precipitate the development of atopic disease. Several other investigators have also documented associations in humans and mice between viral-induced IgE and atopic disease [2, 8, 9].

Probably the strongest associations connecting viral infections to atopic disease have been found in studies looking at the development and exacerbation of asthma [10]. The virus most often associated with atopic disease is respiratory syncytial virus (RSV), a single-stranded RNA paramyxovirus that is a major pathogen in the Northern Hemisphere in the midwinter months and infects nearly all children by 2 years of age [11, 12]. While most children get this infection early in life, a subset of infants (primarily in the 2- to 6-month age range) who become infected with RSV develop a severe bronchiolitis requiring hospitalization. Sigurs et al. [13] showed that after viral infection these individuals were left with an increased risk for developing asthma (odds ratio [OR], 12.7), as well as allergic sensitization (OR, 2.4). The risk remained present through at least 13 years of age [14]. More recently, Wu et al. reported that a large unselected retrospective cohort of children born 4 months prior to the peak of RSV season had both the greatest risk of hospitalization for lower respiratory tract illness and the greatest risk of asthma between 4 and 5 years of age [15]. This study again supports the idea that a severe RSV infection requiring hospitalzation is associated with the development of atopy.

It has been suggested that the risk of atopy is not from the viral infection but instead is a result of an immune alteration that allows for reduced antiviral immunity [16]. In fact, some investigators have shown that individuals with atopic disease have reduced production of IFN-*α* from a subset of their dendritic cells, which may predispose them to a weaker anti-viral immune response [17–19].

If the risk of atopic disease was not imparted by the viral infection, then reducing the severity of the infection with an anti-viral would be expected to have no effect on the subsequent development of atopy. Interestingly, the use of a monoclonal antibody directed against an epitope in the A antigenic site of the F protein of RSV (palivizumab) as prophylaxis against RSV in preterm infants, significantly increased the time to the first episode of recurrent wheeze and physician-diagnosed recurrent wheeze (hazard ratio (HR) = 0.46) [20]. Furthermore, in a study of 175 patients who had acute RSV in which some received ribavirin and some did not, the rates of physician-diagnosed wheezing were decreased, as was the rate of allergic sensitization in the group that received the antiviral medication [21]. Together, these studies suggest that the prevention or early treatment of viral infections may be able to prevent subsequent development of asthma and atopic disease. 

It is important to note that not all studies have shown a risk of RSV infection and atopy. For example, the Tucson Children's Respiratory Study includes more than 800 children with documented RSV infection in infancy. However, unlike the study by Stein et al., these infections were not severe enough to require hospitalization [22]. These children have been followed longitudinally for the development of asthma and other atopic diseases. And while RSV infection was shown to increase the risk of wheezing early in life, it was a transient risk, disappearing between 10 and 13 years of age. 

Another virus associated with atopic disease is human rhinovirus (hRV), a nonenveloped, single-stranded RNA virus of the family Picornaviridae. On the basis of the sheer number of viral exposures, hRV is an important pathogen in the development of acute bronchiolitis in infants. In contrast to the seasonal nature of RSV, hRV infections occur throughout the year and are the main cause of bronchiolitis leading to hospitalization in infants outside of the winter months [23]. Children hospitalized with hRV tend to be older than those infected with RSV and are more likely to have a prior history of wheezing. These children often have more atopic risk factors, eczema, allergic sensitization, and parental asthma than do the RSV children [24, 25]. This difference, however, may simply relate to the ages of the infected children rather than to a specific effect of the virus. Similar to RSV, Kotaniemi-Syrjänen et al. [26] reported that children that were hospitalized with wheezing from hRV during the first 2 years of life were at increased risk of childhood asthma (OR 4.14) when compared to children hospitalized for wheezing with other viruses. Furthermore, in a Tennessee cohort of 90,000 children, an increased risk of asthma was found in children with episodes of bronchiolitis during nonwinter months [27]. Additionally, those with bronchiolitis during a month associated with higher rhinovirus exposure had a 25% greater risk of developing childhood asthma than those who developed infections during months where RSV was the primary cause of bronchiolitis [27].

Two birth cohort studies have identified outpatient hRV wheezing illnesses as important predictors of childhood asthma development as well. The Childhood Origins of Asthma (COAST) study examined a high-risk (1 parent with asthma or allergy) birth cohort of suburban children [28]. This study showed that a symptomatic hRV infection was the most important risk factor for development of wheeze by age 3 [29] and asthma by age 6 (OR, 25.6) [30]. The association of wheezing with prior hRV infection was stronger than that with RSV or any other viral pathogen. The COAST study involved a multivariate analysis that attempted to correct for other factors, such as pets, older siblings, participation in day care, breastfeeding, atopic dermatitis, and the presence of food-specific IgE. Further analysis of cytokine profiles in this cohort suggested that children with impaired Th1 responses (decreased release of interferon-*γ* (IFN-*γ*) from cord blood monocytes) had increased numbers of viral infections in the first year of life [31]. However, it is not clear what role IFN-*γ* (i.e., type II IFN) plays in the development of atopic disease (as opposed to IFN-*α*).

In a similar high-risk birth cohort in Australia, Kusel et al. examined the risk of wheezing at 5 years of age in 198 patients who were at high risk of developing asthma [32]. These patients were enrolled as infants, monitored for respiratory infections, and had periodic checks for the development of asthma and atopy. At 5 years of age, the patients with current wheeze and asthma had a history of higher rates of respiratory infections with hRV or RSV. This association between viral infection and asthma was limited to the subgroup of patients who had developed allergic sensitization before 2 years of age suggesting a role between early-life viral infection and development of allergic sensitization. Indeed, we have previously proposed that the ability of viruses to drive an atopic risk is limited to the first couple of years of life, after which viruses are more prone to exacerbate existing disease rather than lead to the development of new atopy [33].

As in studies of RSV and asthma, there is conflicting data concerning the causal role of viral infection and atopy. Recent statistical modeling of data from the COAST cohort claims that sensitization to aeroallergen primarily precedes any RV-associated wheezing [25], arguing against viral infection being causative in subsequent aeroallergen sensitization. It should be noted that the statistical model used for this study assumed continuous monitoring of both wheezing and sensitization. However, the cohort was only continuously monitored for wheezing and monitored annually for sensitization to aeroallergens. Consequently, while this study supports the findings from the Australian cohort, it does not fully refute the possibility that viral infection could be causative in initial allergic sensitization [34]. The mechanism(s) that might be responsible for translating these viral infections into atopic disease have only recently begun to be elucidated. It is interesting that in a mouse model there is a critical role for anti-viral IgE in the translation of the viral immune response into atopic disease, and this route is dependent upon the function of specific resident dendritic cells in the airway [2, 78].

## 3. The Role for the Resident Dendritic Cell

Dendritic cells (DCs) represent an important class of resident lung cells that are involved in initiating the immune response. While the nomenclature differs slightly between mouse and human, the knowledge and understanding gained from mouse DCs has led to better understanding in the human. In the mouse, DCs have been divided into two broad subtypes—conventional dendritic cells (cDCs) and plasmacytoid dendritic cells (pDCs). The cDCs are the major antigen-presenting cells in the body, whereas pDCs are major producers of the cytokine interferon-*α* (IFN-*α*; type 1 IFN) and to a lesser extent IL12 [35, 36] and have been shown to play an important role in modulating immune responses as opposed to initiating them [37–42]. The cDCs express high levels of the integrin CD11c, whereas the pDCs express Siglec-H, Ly6C, and B220, but low levels of CD11c. However, there is some confusion in the literature over identification of cDC, since other cell types can express CD11c. Recently, the zinc finger transcription factor Zbtb46 was shown to be specifically expressed in human and mouse cDCs [43, 44]. In the future, this marker will likely be used to specifically identify these cells. Conventional DCs can be further subdivided based on expression of the integrins CD103 and CD11b. cDCs expressing CD103 (CD103^+^ CD11b^−^), the *α*E integrin, are resident cells found at mucosal sites under homeostatic conditions and are associated with the respiratory epithelium. These cells project their dendritic extensions between epithelial cells, allowing them to directly sample content of the airway lumen, and have been termed the “intraepithelial” subset [45]. CD11b expressing cDC (CD103^−^ CD11b^+^), on the other hand, is associated with inflammatory stimuli and is found in the respiratory tract during ongoing activation of the immune response [46–48].

In humans, dendritic cells have also been divided into two broad subtypes—myeloid dendritic cells (mDCs—the equivalent of mouse cDCs: CD11c+CD11b+Gr1−B220−) and pDCs (CD11c+CD11b−Gr1+B220+) [49]. Lung DCs in humans have been harder to characterize due to a lack of validated markers and the difficulty in obtaining human lung tissues for investigation; however, as mentioned, the novel transcription factor Zbtb46 is a robust marker of human cDC and will likely help clean up the identification of human DC subsets in the future [43, 44]. 

Dendritic cells are found throughout the lung and the entire respiratory tract epithelium, including the nose, nasopharynx, large conducting airways, bronchi, bronchioles, and alveolar interstitium [45, 50, 51]. These cells form an intricate cellular network just below the epithelial cell layer [52, 53]. Their location and number underlie their function as sentinels and primary responders to potential pathogens. It has been estimated that there are between 400 and 800 resident dendritic cells per mm^2^ of epithelial surface in the rat airway [54]. In the absence of antigen, airway cDCs rapidly turnover, with an estimated 85% of the cell population changing every 36–48 h; antigen exposure greatly increases this emigration. In the rat, it is estimated that the half-life of lung dendritic cells ranged from 1.5–2 days in the airway to 3-4 days in the periphery of the lung—both of which are much less than the 9 days or more in skin [55, 56]. 

Classically, cDCs ingest foreign proteins primarily through attachment of pattern recognition receptors, such as immunoglobulin (FcR), carbohydrate (mannose receptor), complement (CR3), and toll-like receptors (TLRs). Once bound and ingested by pinocytosis, the proteins begin to break down in the endolysosome. These peptides then merge with a major histocompatibility complex (MHC) class II containing vesicle. This allows the peptides to bind to the MHC class II molecules and be exported to the surface, where they are presented to T cells. However, most anti-viral immune responses are CD8 dependent and require presentation of antigen in the context of MHC class I. While some viruses can directly infect dendritic cells (e.g., herpes simplex virus, vaccinia virus, and measles virus) leading to presentation in MHC class I molecules, most do not appear to directly infect the dendritic cell [57–60]. To take up virus without being directly infected and yet express in it terms of MHC class I, the dendritic cell can express virus-binding receptors. For example, human immunodeficiency virus, cytomegalovirus, Ebola virus, dengue virus, and hepatitis C virus are all capable of binding to a C-type lectin, a specific intercellular adhesion molecule nonintegrin on DCs known as DC-SIGN [61]. Since many viruses do not have known receptors and cannot directly infect dendritic cells, the cell must also ingest virally infected cells (and virus) from the extracellular environment to obtain viral proteins to process. Then, through a process known as cross-presentation, the cDC is able to place the viral peptides in the context of MHC class I (rather than just MHC class II) [62]. 

It appears that cross-presentation (i.e., expressing extracellular peptides in the context of MHC class I) is a regular occurrence in DCs, even in the absence of an inflammatory insult. In fact, in this situation, it often initiates tolerance—especially if it occurs in the absence of dendritic cell activation [63, 64]. However, in viral infections, the inflammatory response rapidly leads to activation of cDCs, and the cross-presentation pathway leads to a productive immune response. In the case of influenza or parainfluenza, it has been shown that pulmonary DCs rapidly become activated and migrate to the draining lymph node [65, 66]. Within 6 hours of exposure to the virus, the draining lymph nodes contain numerous activated and mature pulmonary DCs. Interestingly, this DC response in the lymph node seems limited to the first inflammatory antigen encountered in the lung [65].

Plasmacytoid DCs are thought to play a major role in the antiviral immune response through production of IFN-*α* and are rapidly recruited to the airway during respiratory tract viral infections [66, 67]. The airway normally contains a much lower frequency of pDCs, although the numbers of pDCs may be influenced by genetic factors [68]. In a mouse model of viral respiratory infection, few pDCs are noted within the lung parenchyma at baseline, but with viral inoculation pDCs are rapidly recruited from the bone marrow and, within several days, comprise a majority of DCs in the virally infected lung [66, 69]. Through production of IFN-*α*, the pDCs are capable of skewing the immune system toward a predominantly Th1 antiviral immune response. Conversely, in a recent study by Pritchard et al., it was shown that depletion of pDC from RV-stimulated peripheral blood mononuclear cell (PBMC) cultures led to markedly inhibited IFN-*α* secretion and to a significant increase in expression and skewing toward production of Th2 cytokines [70]. 

In short, while the pDC population is responding directly to the lung insult, the cDCs that have ingested the invading organism are maturing in the draining lymph node and presenting antigen to T cells. 

## 4. Putting It All Together

Despite the fact that most inhaled antigens are transported to the lymph nodes by lung-derived DCs, the usual outcome following the inhalation of harmless protein antigen is the induction of tolerance. Sakamoto et al. [71] infected mice with enough influenza virus to cause pneumonia; the mice were subsequently sensitized to aerosolized ovalbumin (OVA) associated with aluminum hydroxide adjuvant following the infection. Influenza infection prior to allergen sensitization resulted in elevated OVA-specific IgE levels—provided that the OVA exposure occurred during the acute infection on days 2–6 after inoculation (p.i.). Exposure to OVA 14 days p.i. failed to enhance allergic sensitization. Further studies confirmed these observations with additional allergen challenge 3-4 weeks after primary sensitization [72, 73]. Moreover, Yamamoto et al. [73] reported a transient increase in airway DCs from day 2 to day 5 of influenza infection. In mice sensitized to OVA aerosol at the time of infection, the increase in DC number persisted for up to 5 weeks and was associated with high MHC class II expression by these cells. The authors concluded that recruitment of DCs to the airways during influenza infection may have contributed to enhanced sensitization to aeroallergens. Similar, to influenza, mice infected with RSV which were exposed to non-viral antigens (ragweed and OVA) with or without alum adjuvant from day 4 to day 8 p.i. were found to produce greater levels of allergen-specific antibodies in serum (IgE and IgG) and bronchoalveolar lavage (BAL) fluid (IgA and IgG) than mice that were not infected with the virus [74, 75].

Sendai virus (SeV), the murine parainfluenza virus type 1, is a paramyxovirus and natural rodent pathogen. In a strain-dependent fashion, mice which survive a severe SeV infection develop chronic airway hyperreactivity and IL-13-dependent mucous cell metaplasia, similar to human infants infected with RSV [76]. We have shown that this translation of viral to atopic disease requires an INF-*α*-dependent accumulation of CD49d^+^ polymorphonuclear neutrophils in the lung, which in turn induces resident lung cDC to express the high-affinity receptor for IgE (Fc*ε*RI) [77]. Expression of Fc*ε*RI on lung cDCs and the subsequent production of anti-SeV IgE were required for the development of postviral atopic disease [78]. In fact, we and others were able to show that crosslinking Fc*ε*RI on the lung cDC led to production of CCL28 and subsequent recruitment of IL-13 producing Th2 cells in an antigen nonspecific fashion [78, 79].

Further, we have shown that a single exposure to a nonviral antigen (OVA) during an active anti-viral immune response is sufficient to drive significant and marked IgE production against the non-viral antigen [2]. Unlike previous reports with RSV or influenza, the development of OVA-specific IgE during an SeV infection did not require the use of an adjuvant. A subsequent challenge with the antigen alone led to augmented airway hyperreactivity and mucous cell metaplasia, demonstrating the development of atopic disease, as a result of a severe respiratory viral infection. 

While these initial observations were made in mice, we have documented that the cDC Fc*ε*RI-CCL28 axis appears operative in humans [80]. Further, it has been shown that expression of Fc*ε*RI on human peripheral blood cDC is increased by respiratory viral infection [9]. However, unlike in the mouse, human cDCs appear to express Fc*ε*RI from birth, although the regulation of its expression does appear to be quite complex with low levels of IgE having less of an effect on Fc*ε*RI levels compared to higher IgE concentrations [81]. Therefore, while not completely validated in the human, critical portions of the cDC Fc*ε*RI-CCL28 pathway appear intact in both mouse and man and begin to provide us with one mechanistic explanation for the translation of viral into atopic disease. 

The process of inhalational tolerance is also influenced by pDCs, and there are good studies to suggest that these cells also play a role in the development of postviral atopic disease. In mice in which pDCs were depleted, RSV infection led to greater disease with increased viral titers and airway inflammation, as well as postviral airway hyperresponsiveness [69, 82]. This outcome is consistent with the need for effective IFN-*α* production to control the infection and the fact that pDCs are a major cellular source of IFN-*α* during a viral infection. Therefore, a reduction in the ability to release IFN-*α* could be a risk for developing atopic disease. In fact, studies of pDCs from peripheral blood and bronchoalveolar lavage (BAL) have documented that patients with allergic asthma have diminished influenza-induced and rhinovirus-induced IFN-*α* secretion, respectively, from pDCs when compared with healthy control subjects [19, 83]. Tversky et al. [84] also found a similar impairment of IFN-*α* responses in pDCs from allergic subjects after stimulation with oligodeoxynucleotide-containing unmethylated CpG motifs (TLR9 agonist). Interestingly, there appears to be a counterbalance in human pDC when it comes to IgE- and TLR9-mediated IFN-*α* production. Cross-linking Fc*ε*RI on human pDC was associated with reduced TLR9 expression and subsequent IFN-*α* production from a TLR9 agonist, while exposure to a TLR9 agonist first led to reduced expression of Fc*ε*RI in the cells [85]. Furthermore, in an analysis of children from the COAST cohort, Fc*ε*RI*α* expression on pDCs was inversely associated with hRV-induced IFN-*α* and IFN-*λ*1 production [86]. The allergic asthmatic children in this study had higher surface expression of Fc*ε*RI*α* on pDCs, and after Fc*ε*RI cross-linking, they had significantly lower hRV-induced IFN responses than the allergic nonasthmatic children.

## 5. Conclusion

**Figure 1:**
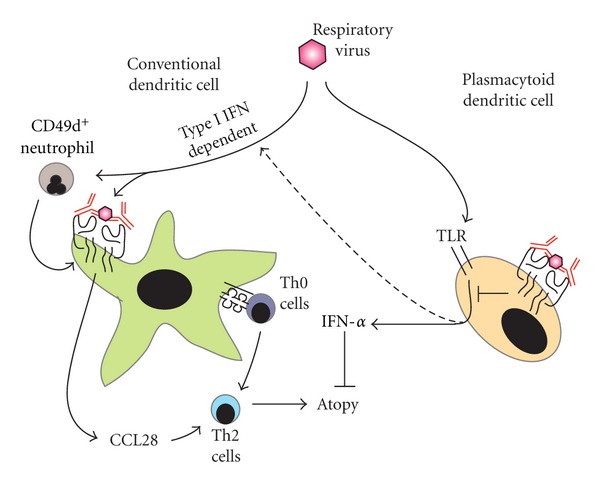
Proposed role of resident lung dendritic cells in the development of postviral atopic disease. After infection with a respiratory virus, both conventional (green cell) and plasmacytoid (tan cell) dendritic cells can influence the development of atopy. Conventional dendritic cells (cDCs) are able to present antigen to naïve T cells and skew their development towards a Th2 lineage (purple cell being converted to the blue cell). In addition, in a type I IFN-dependent fashion, CD49d-expressing neutrophils are recruited to the airway during the infection. These cells induce Fc*ε*RI expression on lung cDC, which can be bound by anti-viral IgE. Subsequent cross-linking of the anti-viral IgE by virus leads to the production of CCL28, which further recruits Th2 cells to the airway, leading to atopic disease. The plasmacytoid dendritic cell (pDC) is induced to produce IFN-*α* by the viral infection through toll-like receptors (TLRs). The IFN-*α* blocks the development of atopy. Whether this type I IFN drives the CD49d^+^ neutrophil response is not known (the dotted line). Similar to the cDC, anti-viral IgE can bind Fc*ε*RI on the pDC, with the free virus cross-linking the receptor. This impairs the release of IFN-*α* from the TLR removing the pDC-mediated inhibition of postviral atopic disease. See text for more details.

While the exact role viruses play in the overall development of asthma and atopic disease remains controversial, the data are compounding to suggest that resident lung cDC and pDC play a critical role in this process. Specifically, as shown in Figure 1, cDCs play a major role in both aeroallergen sensitization and in recruiting Th2 cells in an IgE-dependent fashion. These Th2 cells then lead to the development of postviral atopic disease with subsequent IgE production leading to spreading of allergic sensitization (something we have referred to as an “atopic cycle” [33]). Likely it is the combination of a reduced pDC IFN-*α* response in the presence of an activated cDC-Fc*ε*RI-IgE-CCL28 axis that leads to the development of postviral atopic disease. Of course, it is also quite likely that an individual's genetic factors contribute to their overall response to the viral infection, further complicating studies exploring the risk of atopic disease and viral infection. Nonetheless, these studies have provided insights that will help to guide future therapeutic interventions to alter the pulmonary resident DC response and prevent the translation of viral into atopic disease. In fact, we hope it will not be long before infants who have severe viral infections will be treated with medications designed to block the development of postviral atopic disease.
